# Development and implementation of industrialized, fully automated high throughput screening systems

**DOI:** 10.1155/S1463924603000105

**Published:** 2003

**Authors:** Benjamin G. Carvalho

**Affiliations:** Bristol-Myers Squibb Wallingford CT USA

## Abstract

Automation has long been a resource for high-throughput screening at Bristol-Myers Squibb. However, with growing deck sizes and decreasing time lines, a new generation of more robust, supportable automated systems was necessary for accomplishing high-throughput screening goals. Implementation of this new generation of automated systems required numerous decisions concerning hardware, software and the value of in-house automation expertise. This project has resulted in fast, flexible, industrialized automation systems with a strong in-house support structure that we believe meets our current high-throughput screening requirements and will continue to meet them well into the future.


Journal of Automated Methods & Management in Chemistry
Vol. 25, No. 3 (May–June 2003) pp. 63–66

Taylor & Francis

Taylor & Francis Group

### Development and implementation of industrialized, fully automated high throughput screening systems†

**Benjamin G. Carvalho***
*Bristol-Myers Squibb, Wallingford, CT, USA*

*Automation has long been a resource for high-throughput screening at Bristol-Myers Squibb. However, with growing deck sizes and decreasing time lines, a new generation of more robust, supportable automated systems was necessary for accomplishing high-throughput screening goals. Implementation of this new generation of automated systems required numerous decisions concerning hardware, software and the value of in-house automation expertise. This project has resulted in fast, flexible, industrialized automation systems with a strong in-house support structure that we believe meets our current high-throughput screening requirements and will continue to meet them well into the future.*

### Introduction

The past few years have seen tremendous advances in the areas of assay miniaturization and laboratory automation technology. Because of the steady increase in screening numbers, accompanied by a steady decrease in desired time-lines, it was decided that we must revamp our old screening infrastructure so that much of this new technology could be incorporated into our screening structure. In addition, given the current pace of change, it was felt that we must construct a system that could accommodate future technologies as well. What I would like to cover here are the decisions that we made during the construction of our systems and how we made them, starting with the overarching principle of laboratory automation that we used to guide our process. I will cover required system capabilities, strategic decisions, including development of software, hardware and data-handling systems, and I will look at the impact that these decisions have had on our high-throughput screening operation.

### User-FIRST

We began this system redesign project in early 2001 with a guiding principle that we would use to help orient our decision-making process. That is the User-FIRST principle, with the Users being our high-throughput screening automation clients and FIRST being an acronym for Flexibility, Integration, Reliability, Support and Throughput.

The initial tenet of our principle is that system design must be user oriented. It is no good designing a system that does not fulfil the requirements of our HTS clients. This means that a core group of automation-savvy users would be involved in every automation project, from design and prototyping to final build, also with responsibility for testing and support.

The FIRST portion encompasses what we feel are five requirements for any automated HTS system.

- Flexibility: a successful system design must be able to support multiple assay workflows, potentially in parallel. It must also be able to incorporate easily new instrumentation or new sample formats.
- Integration: a good design will be modular, standardizing both hardware and software interfaces between components and systems.
- Reliability: systems must be capable of running unattended. Therefore, systems should be extensively tested before release. Errors should be forced, with error recovery behaviour observed. Redundant capabilities will be built in as a fall back strategy. Industrialized automation hardware will be used whenever possible. System peripheral equipment should be chosen based on both capability and durability.
- Support: hardware and software support are key to keeping systems up and running. As such, an experts trained support team should be in place. Additionally, comprehensive preventive maintenance procedures should be put into place.
- Throughput: the throughput of an automated laboratory system should be limited by only scientific factors, biology, chemistry or physics, not by instrumentation.

### System specifications

Through work with our user community, we were able to cull efficiency requirements for a ‘universal’ HTS system. This system would be the blueprint upon which all of our systems would be based.

The requirements for the new system deal with overcoming the four main bottlenecks seen with our older systems. These bottlenecks were robot speed and reliability, sample transfer time, data handling and error recovery. Robot speed is crucial, as these new systems must be able to add plates into the system without robot time being the limiting factor. Ideally, we would like to see screens with pace times of 5 min or less. Sample transfer time is also important. To keep pace times to a minimum, the new systems should be able to accomplish

*e-mail: carvalho@bms.com
†This paper was initially presented at the ISLAR 2002 Conference and is reproduced here by kind permission of Zymark Corporation.

384-well transfers on a scale of 2–3 min. Data handling was all done external to the system and required hours of work. Any new system should have an integrated data-handling component, one that would reduce and report data in real time. Error recovery on old systems was difficult at best, impossible at worst. In addition, it required the presence of a system designer. This inability to recover errors led to a loss of time, reagent and compound.

Once the parameters for the system had been established, we had to begin to answer some questions. The first was whether to build these systems internally or to have them built through an external collaboration. There are advantages to both approaches. External development offers a speed advantage. By contracting out to a group that has previously done similar jobs, we should see results sooner. This would result in a quicker impact on our screening community. However, there are also advantages to internal development. The first is cost. Buying individual parts and building the system ourselves was shown to be cheaper than purchasing a prebuilt system. Second, and more importantly, designing and building in-house allows us to develop internal expertise. We would then have the required knowledge in our staff to build, rebuild, improve or repair systems. Weighing both options, the internal development, due to the development of in-house expertise, brought us closer to our User-FIRST goals.

After deciding to build in house, the next decision centred on our control software. We had two options: creating our own software or purchasing a commercially available product. Writing our own software would guarantee a package directed towards our needs, however, we would be reinventing a very big wheel. At the time there were a number of commercially available products that offered the range of speed and flexibility that we were looking for. After a careful search process, our final decision was to go with CRS Polara.

CRS Polara software was chosen for the simple reason that it best met all of our previously stated User-FIRST criteria. The users see a standard interface that works in the same manner for all systems, large or small. Additionally, this interface is easy to use, as method development is simply a matter of dragging and dropping unit operations in the correct order. Polara also has instrument interfaces that are similar in appearance and use for all peripherals, regardless of the instrument.

In terms of flexibility, Polara allows for non-linear workflows, meaning that plates can move from any one place to any other, depending entirely on the method. It is also capable of supporting parallel assays, and adding new instrumentation is a fairly simple matter. In terms of integration, CRS had already integrated over 100 instruments from a number of different vendors, allowing us to pick and choose which instruments we felt were the most robust and had the best applications for our assays. Additionally, CRS has an open integration architecture, allowing us to write device drivers for other instruments not already integrated. This would also include the ability to integrate custom hardware if necessary. We also felt that the reliability of the CRS system would be more than acceptable due to the presence of the industrial CRS robot.

Going with CRS allows us to build a strong support structure through a combination of the offered CRS courses in preventive maintenance, user level interaction and instrumentation driver writing. Additionally, the ability to use instruments that were proven to be durable decreased the support burden for system peripherals. Finally, the software scheduler showed the ability to schedule plates at intervals much lower than our original goal of 5 min per plate.

### Hardware

The hardware on one of our HTS screening systems breaks down into five basic categories: robot arm, liquid handlers, incubators, plate readers and other peripherals (figure 1). The robot is a CRS T475 track and robot, integrated with the CRS software. It is an industrial robot with six axes of rotation on the arm and a seventh track axis.

We have two types of liquid handlers on the system: non-contact bulk reagent dispensers, capable of 96 or 384 well addition, and 384 tip-based devices, capable of both 384 and 1536 dispensing, used for sample transfer or reagent addition, if necessary. There are numerous (six or more) bulk reagent dispensers per system, while transfer devices are generally kept to one or two.

There are also two types of incubators on the system. One is for open air, room temperature incubation, while the other has active temperature and $CO_2$ control with passive humidity. The temperature range on this incubators is from room temperature to over 37°C. These incubators are ideal for automating cellular assays.

We also have a large variety of plate readers on our different systems. Depending on the type of assay being run, we can read fluorescence, luminescence, absorbance, SPA, TRF or kinetic reads.

Finally, there is peripheral hardware. These pieces include a plate rotator, a delidder, a sealer, a barcode reader and a shaker. Not shown in figure 1 are a few pieces that add robustness to the system. We have plumbed all of our tables with air, DI water and $CO_2$ from house supplies. Also, all waste is plumbed off of the system to house drains or collection points if necessary. In this manner, all facilities can be supplied to the system without interruption. Finally, all systems are wired to our building supplied UPS power, getting us past short interruptions in power and allowing the opportunity for a graceful shutdown when necessary.

### Data handling and paging

One of the bottlenecks that we wanted to overcome with our new generation of HTS robots was that of data handling. What we found with our older systems is that our screeners were spending half their time reducing data and entering it into the database. We were now planning on building systems that could generate data points at a

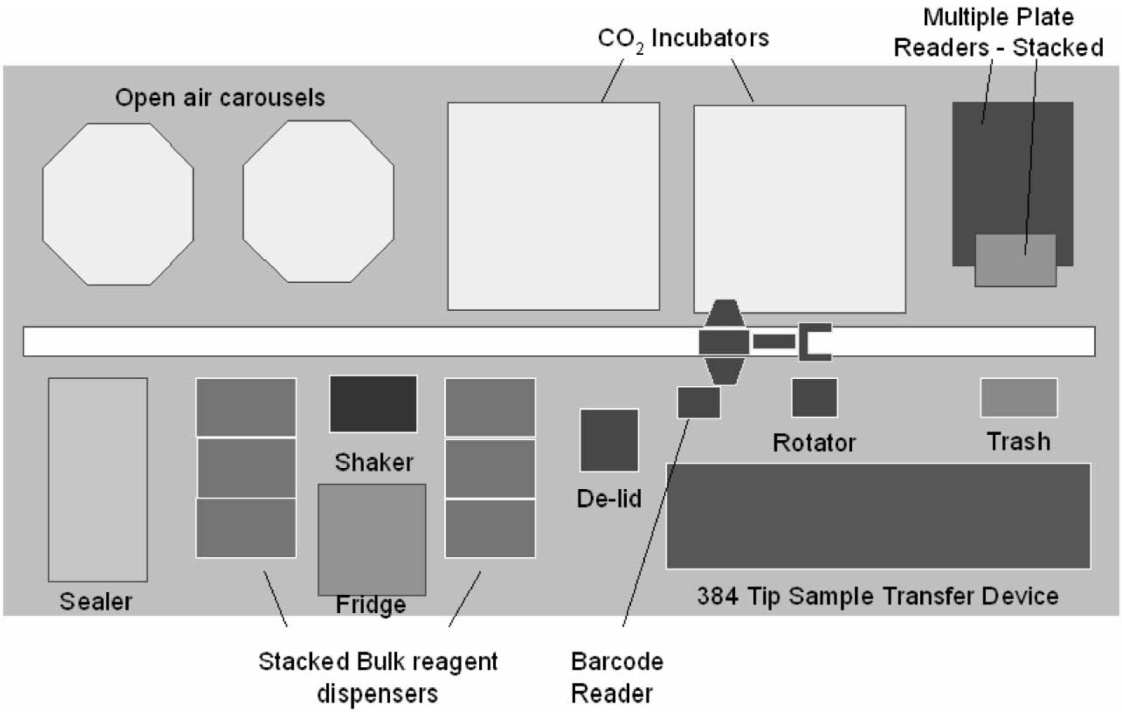

*Figure 1. Universal Screening Bench.*

vastly increased rate. However, even if we could generate tens or hundreds of thousands of datum points a day, if we did not have the ability to reduce them to meaningful data in a short period, there was no use in having the screening capacity. What we have built in-house is a system called QCMAD.

QCMAD, an acronym for Quality Control Monitoring/Automated Data load, allows for automatic data reduction and upload to the screening database of data generated on a Polara system. We have been able to integrate Polara seamlessly with QCMAD thanks to the open structure of the Polara data extraction software. This data extraction software pulls plate data out of the Polara run-generated log file. This program was supplied in open Visual Basic code, allowing us to alter the format that the data was reported in as well as the amount of information known to the system at run time. The result of these alterations was to present data in a single format into the QCMAD program, while the system was running, regardless of the plate reader being used. Once this is accomplished, QCMAD takes over. Over the course of a run, QCMAD monitors control and blank data on each plate to determine the value of the data coming off the system. If data values go outside of established parameters, the system user is paged. Data that are accepted are reduced and uploaded. The user is then able to access plate and well information through a series of web-based charts. These web-based charts give users access to plate means, signal window, signal/background ratio, signal/noise ratio, CV and $Z'$. Data that are accepted are uploaded to the main database where they are automatically integrated with plate map information.

Building this data-handling system in-house has allowed us to design a system that was tailored to our needs. We have built up the expertise to keep this system running, and in the event that changes to the system become necessary, we have the in-house support staff to make those changes happen.

The same program that feeds data from the screening system to QCMAD also monitors the system for any errors or warnings that may occur. These messages are then sent to a paging server, which sends pages to users and to support personnel. These people are paged when system intervention is necessary, when a system warning occurs or when the system is complete.

### Impact on screening

This project is now almost 2 years old. In that time, we have built six HTS systems with the capabilities discussed above. We are also beginning to branch out to smaller workstation-type screening systems. We feel that applying the User-FIRST principle to our system design has allowed us to achieve the success that we have had so far. We have been able to make a vast number of improvements over our previous capabilities, and we have left ourselves the room, and generated the expertise, to make further improvements as technology advances.

As far as the user is concerned, all of our CRS systems look and act alike. With a few exceptions they have the same hardware, and interfaces to all instruments look similar. Additionally, data upload and reduction is as easy as a few clicks on a web page.

Looking at system flexibility, we see a number of changes. Old systems were screen specific. Any changes in workflow required reprogramming and revalidation that would take days or weeks. Our new systems can easily support multiple types of workflows, running different workflows in parallel, if necessary. We can move plates from any one instrument to any other. Finally, programming of new methods takes only minutes, and addition of new instrumentation is usually done in as little as an hour. Also, by building our systems off a universal pattern, screens are not tied to a single system.

Concerning the integration part of our User-FIRST principle, we now have multiple systems with identical user interfaces, and we have no significant restrictions on the types of instruments to be used on these systems. To this point, we have not had a single instrument that has not been integrated.

With the new systems, reliability has increased dramatically. With the addition of industrialized robotic CRS arms, and with the use of a thoroughly tested control software package, we are easily capable of running 24/7, with system failures being exceedingly rare. Additionally, most failures that due occur are due to GRBH (Good Robot, Bad Human), and are easily recovered.

Our available support for HTS systems has become another source of confidence. Because both the screening systems and the data-handling system were built in house, qualified support personnel are always on hand. Changes or repairs to the systems can be done the day they are needed. Additionally, the knowledge is in place to build more systems or extend the technology by writing drivers to custom equipment, if necessary. Finally, the openness of CRS software has allowed us to integrate a paging system for alerting users and support staff of system and data status.

Lastly, throughput with the new systems has improved dramatically. Addition of sample transfer devices and of non-linear processing has allowed us to cut pace times from 12 min per plate to as low as 2.5–3 min per plate. This decrease in pace time, along with the ability confidently to run 24/7 has increased our throughput by at least a factor of eight. The addition of QCMAD has also increased throughput in that data are now monitored in real time and uploaded to the database much more quickly than in the past.

### Conclusion

Applying the User-FIRST principle to building our high throughput screening systems has paid off in may ways. We feel that the advantages of in-house system construction far outweigh the additional time necessary to learn the new technology. We now have highly flexible, easy-to-use HTS systems. Our in-house personnel are capable of solving most of our support issues. The open architecture of the CRS software has allowed us to integrate paging and data handling components to these systems. Throughput has been increased by at least a factor of eight. The industrialized systems are very reliable, leading to far fewer failed runs, meaning less loss of time, reagent or sample. Finally, the open architecture and the CRS philosophy of integrating equipment from any company should allow these systems to fulfill our screening needs far into the future.

### Acknowledgements

The following institutions and personnnel are thanked: Automation Technologies: Alastair Binnie, Jim Myslik, Joe Yacobucci, Yvonne FitzGerald, Chris Strom, Juan Cadavid, Gary Jones; ITG: Chris Bernard, Jim Mongillo; Core Automated Screening: Steve Innaimo, Jonathan O'Connell, Sandra Armento, Kim Marmora, Jennifer Zewinski; Facilities: Martin Goodness, Dennis Baneck, George Krom; Discovery Informatics: Dan Maloney, Nelly Masias.

